# Oxaluria—II

**Published:** 1908-10-10

**Authors:** 


### Medicine.

### OXALURIA—II.

It is clear from what has already been said that one of the factors capable of the greatest variation as regards calcium oxalate crystals in the urine is the vegetable dietary. Vegetables are the main source of exogenous oxalates, which pass through the system unaltered. Weight for weight, cocoa, tea, spinach, and rhubarb come high in the oxalate scale, whereas potatoes, cabbage, and coffee come much lower. A person who drinks much tea and is fond of spinach and rhubarb is much more likely to have an abundance of calcium oxalate crystals in the urine than is one who drinks coffee instead of tea, and who is for the most part content with potatoes and cabbage as his daily vegetables.

The nature of the drinking-water as regards its hardness, or the reverse—especially in regard to the relative as well as to the absolute quantities of calcium and magnesium it contains—may be a factor of considerable importance in the production of oxaluria in the clinical sense. It is true that man absorbs but little of the inorganic calcium that he ingests, or, at any rate, if he absorbs it he excretes the greater part of it back into the bowel again; nevertheless he does absorb some of it, and the greater the proportion of calcium in the urine the greater the probability of calcium oxalate crystals being precipitated, especially if the amount of magnesium present is comparatively small.

Meat contains much less salts of oxalic acid than do vegetables, and it does not modify the total quantity of oxalates in the urine to any great extent; nevertheless, there is one way in which meat diet tends to increase the numbers of calcium oxalate crystals in the urine, and that is by increasing the acidity of the latter. It is possible for the crystals to come down in a neutral or even in an alkaline urine, but they are more commonly found in urines that are acid. Meat diet increases the acidity of urine, and thus tends to augment the precipitation of envelope crystals. An herbivorous animal has an alkaline urine; so also has a strict vegetarian. A vegetarian is likely to pass much larger total quantities of oxalates than is a person who eats mixed diet; but it is quite possible that his urine, owing to its alkalinity, may contain fewer oxalate crystals of the two.

Concentration being a factor in the precipitation of oxalate of lime crystals it is clear that summer urines are more likely to exhibit them than are winter urines. For the same reason oxaluria may be found temporarily after a Turkish bath, or after any prolonged exertion accompanied by sweating. In febrile states it might have been expected that oxaluria would be observed owing to the concentration of the urine; but this is by no means always the case, largely, no doubt, owing to the patient no longer partaking of vegetables containing exogenous oxalates. The effects of disease upon endogenous oxalates are only beginning to be studied, and little or no useful information upon this aspect of the matter can yet be given; but that something serviceable will presently be discovered in this direction seems likely, when Mayer is able to report that tuberculous patients with pyrexia excrete greatly increased quantities of oxalates—0.05 gram per diem instead of the normal 0.015 gram; and that there is absolute oxaluria in purulent meningitis, erysipelas, and septicæmia, whereas this is not the case in measles, scarlet fever, diphtheria, or typhoid fever.

One point which it is very important to remember in connection with concentration of the urine is this: the sediments are obtained by complete centrifugalisation, after which the supernatant fluid is poured off, leaving only the centrifugalised deposit and a few drops of urine. The latter is of such small bulk that evaporation is apt to lead too readily to the precipitation of crystals that were not present in the urine before it was centrifugalised. The discovery of minute crystals in such a deposit is a matter of no moment. The freshly formed crystals are sure to be very small, whereas those that were present in the original urine would be comparatively large; it is nearly always advisable to note the approximate sizes of the crystals seen. Dr. W. G. Smith points out that it is often possible to be sure that a urine contains excess of crystals of oxalate of lime, even without microscopical examination, if the specimen, having stood in a conical glass, exhibits to the naked eye: (1) Fine white striæ, like scratch lines, along the sides of the glass, produced by deposition of crystals along roughnesses and scratches. (2) A hummocky, undulated, but sharply-defined surface on the white stratum of deposit at the bottom of the vessel.

Calcium oxalate crystals are often present in abundance in the urines of perfectly healthy persons. It has been stated that one particular variety of dyspepsia, sometimes attributed to functional nervous trouble, is constantly associated with oxaluria, but it is very difficult to be sure that there is any direct connection between the oxaluria and the dyspeptic symptoms in these cases.

There are, however, at least three ways in which the occurrence of calcium oxalate crystals in the urine is of considerable importance, and these are:

(1) *In Confirmation of Urinary Calculus.*—There may be hosts of calcium oxalate crystals in a urine without any calculus having been formed; but as time goes on, if they persist, some will agglomerate and a stone will begin to grow. The urine in such cases may exhibit nothing pathognomonic; but if in a suspicious case the deposit contains an abundance of oxalate crystals and of red blood corpuscles, the condition is almost certain to be one of mulberry calculus.

(2) *As a Cause for Excess of Nucleoproteid.*—The irritation set up by the presence in the urine of excess of oxalate crystals may produce no symptoms that the patient complains of, and yet it may lead to the urine containing excess of mucus, excess of leucocytes, excess of nucleoproteid, and in a man an abundance of spermatozoa. The nucleoproteid may be mistaken for albumen unless considerable care is taken, and the patient's condition may in consequence be diagnosed as something much more serious than it really is.

(3) *Nocturnal Incontinence.*—The irritation just mentioned may, in a boy or girl, have a very troublesome manifestation in nocturnal enuresis. The latter is, if course, not always due to oxaluria, but in some cases discovery of the latter, followed by steps to cure it, result in the cure of the enuresis at the same time. Occasionally the irritation passes on to an absolute urethritis, and great care may be needed not to attribute the latter to some more common cause.

Oxaluria by no means necessarily requires any treatment at all. The chief circumstances under which active measures may be required to diminish oxaluria will be either when there are signs of irritation of the urinary passages without calculus, in some cases of nocturnal enuresis, and in cases of mulberry calculus, particularly when a stone has been removed and it is desired to prevent the formation of another. The methods of treatment become obvious when the ways in which oxaluria come about are known. They are:

(*a*) To be careful that the vegetables eaten are such as contain a minimum of oxalates; (*b*) to ensure that the urine is not too concentrated, by giving plenty of fluids to drink; (*c*) to prevent the urine being too acid, bicarbonate of soda possibly being required by the mouth; (*d*) to ensure that the proportion of magnesium in the urine is not too low, by giving magnesium sulphate in mildly aperient doses.

